# A Real-World Evidence-Based Study of Long-Term Tyrosine Kinase Inhibitors Dose Reduction or Discontinuation in Patients with Chronic Myeloid Leukaemia

**DOI:** 10.3390/pharmaceutics15051363

**Published:** 2023-04-28

**Authors:** Alicia Martín Roldán, María Del Mar Sánchez Suárez, Carolina Alarcón-Payer, Alberto Jiménez Morales, José Manuel Puerta Puerta

**Affiliations:** 1 Servicio de Farmacia, Hospital Universitario Virgen de las Nieves, 18014 Granada, Spain; 2 Unidad de Gestión Clínica Hematología y Hemoterapia, Hospital Universitario Virgen de las Nieves, 18014 Granada, Spain

**Keywords:** chronic myeloid leukaemia, tyrosine kinase inhibitors, treatment-free remission, molecular recurrence-free survival, low dose, adverse event, real-word evidence

## Abstract

The therapeutic approach to chronic myeloid leukaemia (CML) has changed in recent years. As a result, a high percentage of current patients in the chronic phase of the disease almost have an average life expectancy. Treatment also aims to achieve a stable deep molecular response (DMR) that might allow dose reduction or even treatment discontinuation. These strategies are often used in authentic practices to reduce adverse events, yet their impact on treatment-free remission (TFR) is a controversial debate. In some studies, it has been observed that as many as half of patients can achieve TFR after the discontinuation of TKI treatment. If TFR was more widespread and globally achievable, the perspective on toxicity could be changed. We retrospectively analysed 80 CML patients treated with tyrosine kinase inhibitor (TKI) at a tertiary hospital between 2002 and 2022. From them, 71 patients were treated with low doses of TKI, and 25 were eventually discontinued, 9 of them being discontinued without a previous dose reduction. Regarding patients treated with low doses, only 11 of them had molecular recurrence (15.4%), and the average molecular recurrence free survival (MRFS) was 24.6 months. The MRFS outcome was not affected by any of the variables examined, including gender, Sokal risk scores, prior treatment with interferon or hydroxycarbamide, age at the time of CML diagnosis, the initiation of low-dose therapy and the mean duration of TKI therapy. After TKI discontinuation, all but four patients maintained MMR, with a median follow-up of 29.2 months. In our study, TFR was estimated at 38.9 months (95% CI 4.1–73.9). This study indicates that low-dose treatment and/or TKI discontinuation is a salient, safe alternative to be considered for patients who may suffer adverse events (AEs), which hinder the adherence of TKI and/or deteriorate their life quality. Together with the published literature, it shows that it appears safe to administer reduced doses to patients with CML in the chronic phase. The discontinuation of TKI therapy once a DMR has been reached is one of the goals for these patients. The patient should be assessed globally, and the most appropriate strategy for management should be considered. Future studies are needed to ensure that this approach is included in clinical practice because of the benefits for certain patients and the increased efficiency for the healthcare system.

## 1. Introduction

Chronic myeloid leukaemia (CML) is a disease characterised by the excessive proliferation of granulocytes without compromising their ability to develop into specialised cells. One-fifth of all adult leukaemia cases are caused by CML [1], which is in turn caused by the Philadelphia chromosome, i.e., the reciprocal translocation of chromosomes 9 and 22, t(9; 22)(q34; q11.2), leading to BCR::ABL1 tyrosine kinase [2].

The first treatments for CML consisted of arsenic, radiation therapy of the spleen [3], busulfan, an alkylating agent [4], or the ribonucleotide reductase inhibitor, hydroxyurea. Although these two latter ones could generate hematologic remission, the elimination of Ph+ cells in patients’ bone marrow was really infrequent. The first drug that was able to increase the rate of hematologic remission and induce a partial or complete cytogenetic response was interferon-alpha (IFNα), achieving an improvement in long-term prognosis [5]. In most cases, allogeneic hematopoietic cell transplantation (HCT) was shown to be successful in eliminating Ph+ cells. First, when donors were obtained from an identical twin, and later, when they were extracted from siblings with compatible human leukocyte antigens (HLA) or from HLA-matched allogeneic donors [6].

Allogeneic HCT is considered to be the curative treatment for CML; however, finding a suitable donor is the most problematic factor. Nowadays, both the discovery of imatinib mesylate (IM) as the first designed BCR::ABL1 tyrosine kinase inhibitor (TKI), as well as research performed concerning second (dasatinib, nilotinib and bosutinib) and third-generation (ponatinib) TKIs, has dramatically enhanced the survival expectancy among CML patients [7]. Novel treatments have shown to improve the long-term outcomes among patients with higher rates of deep molecular response (DMR), defined as residual BCR::ABL1 transcript levels of, at least, molecular response log 4 (MR4) on the international scale (IS) [8].

Although they belong to the same pharmacological group, each TKI has different rates of efficacy, AEs and effectiveness against BCR::ABL kinase domain mutations. Therefore, different factors should be considered when initiating and deciding the ideal treatment with TKIs [9]. TKI therapy was initially thought to be lifelong, but data from clinical trials of patients using these first- and second-generation drugs show that approximately 50% of CML patients with sustained minimal residual disease (MRD) can stop receiving TKI therapy without the risk of suffering a molecular relapse, thus finally achieving a treatment-free remission (TFR) [10].

It is worth mentioning that long-term exposure to TKIs has been associated with adverse effects and has a negative impact on the life quality of patients. In addition, there is increasing evidence that second- and third-generation TKIs can potentially cause significant morbidity and mortality [11]. Moreover, long-term treatment with TKIs is significantly costly for society. An optimal disease outcome is normally expected for patients with CML, yet adverse effects should not be disregarded and must be taken into account since they are always plausible. The results of multiple prospective trials have demonstrated that patients who maintain a DMR treatment combined with TKI for at least 2 years may be eligible for trials of TKI discontinuation [10,11].

## 2. Materials and Methods

### 2.1. Overall Study Aim

The present study is a single-centre retrospective cohort analysis of adult CML patients treated with lower doses of TKIs (compared to those of the standards) or with toxicity-related drug discontinuation. The reason patients were treated with TKIs at reduced doses was either because of toxicity, AEs or them having a good response to the treatment. All patients in the chronic phase and treated with imatinib and/or a second-generation TKI (2GTKI), i.e., dasatinib, nilotinib and bosutinib, or a third-generation TKI (3GTKI), i.e., ponatinib, were included in the study. The aim of the study was to evaluate the safety and effectiveness of TKI discontinuation or a low dosage (LD) treatment in maintaining a response.

### 2.2. Study Design

We retrospectively analysed the outcome in real-world TKI dose reduction (DR) or discontinued patients treated at a tertiary level hospital from January 2002 to October 2022 who underwent an imatinib, a 2GTKI and/or a 3GTKI treatment.

The discontinuation of the treatment or switching from a first to a second generation TKI, in most patients, was due to comorbidities, toxicity or a loss of response. The clinical decision was based on the molecular response, tolerability of the current TKI and potential risks of switching to an alternative TKI.

The present study was conducted with the approval of the Ethics and Research Committee of the Andalusian Health Service and in accordance with the proper clinical practices and ethical principles for medical research established by the World Medical Association Declaration of Helsinki.

All patients over 18 years old and diagnosed with chronic phase CML who were treated with TKI at reduced doses or who started the treatment at low doses were included. Patients who discontinued the treatment were also included. A molecular response was not considered for the inclusion of patients in this study. The reason patients were treated with TKIs at reduced doses or a discontinued treatment was toxicity, AEs or them having a maintained response. In the case of discontinuation, all the patients who had not been given the treatment for at least 6 months were included in the study. They were not excluded from the analysis if they stopped having a response before 6 months. This period of follow-up was determined on the basis of several studies that showed that the incidence of molecular recurrence is highest in the first 6 months [12,13]. As the literature regarding their effectiveness on low-dose patients is more limited, all patients on low-dose TKIs were included, regardless of the duration of the dose. All patients included in the study signed the individual informed consent form.

Variables recorded for each individual patient were: socio-demographic (gender and age at diagnosis), risk scores (i.e., Sokal), BCR::ABL1 transcription type, all previous treatments of TKI (including interferon-alpha, cytarabine, busulfan and hydroxycarbamide), the duration and dosage of each TKI treatment, and finally, reasons for dose reduction and the best response to each treatment.

Low doses of TKIs were defined as imatinib < 400 mg daily, dasatinib < 100 mg daily, nilotinib < 600 mg daily, bosutinib < 400 mg daily and ponatinib < 45 mg daily. The molecular monitoring and classification of responses were defined according to current ELN recommendations [8]: in particular, DMR as MR4 (BCR::ABL1 ratio ≤ 0.01% with at least 10,000 copies of ABL1), MR4.5 (BCR::ABL1 ratio ≤ 0.0032% with at least 32,000 copies of ABL1), MR5 (BCR::ABL1 ratio ≤ 0.001% with at least 100,000 copies of ABL1) or CMR (BCR::ABL1 negative). Safety and tolerability evaluations included the incidence and severity of AE, which were reviewed and recorded during each visit in their medical observation phase.

### 2.3. Statistical Analysis

The distribution characteristics of data were first confirmed. For normally distributed data, the mean and standard deviation (SD) were used to describe the central tendency and dispersion of the data, while for non-normally distributed data, the median (M), 25% percentile (P25) and 75% percentile (P75) were used.

We used a Chi-square test (χ^2^) or Fisher’s exact test for categorical variables and Student’s t test or Mann–Whitney test for continuous variables as appropriate after checking for normality with the Shapiro–Wilk test.

Baseline demographic and clinical variables were compared using the aforementioned methods. Clinical variables possibly associated with molecular outcomes after DR were also evaluated using a log-rank test. A *p*-value < 0.05 was considered to be statistically significant.

The following clinical characteristics were evaluated: previous IFNα treatment prior to TKI therapy, median age at diagnosis, Sokal score, duration of TKI therapy and LD TKI therapy.

Molecular recurrence-free survival (MRFS) was defined as the probability of survival in maintained Major molecular remission (MMR) on low doses of TKI and was estimated via the Kaplan–Meier function, where events were MMR loss or increase in TKI dose/change to alternative TKI after MR4 loss. The patients who restarted TKI after losing MR4 were not included in the analysis. Treatment-free remission (TFR) was defined as the probability of survival in maintained MMR while therapy was not given and was estimated via the Kaplan–Meier function, where events were MMR loss. Estimates are provided with 95% confidence intervals (CIs). All statistical tests were performed using R^®^Commander software (version 2.8-0).

## 3. Results

### 3.1. Baseline Characteristics of CML Patients Treated with TKIs

Of all the patients treated with TKI, 80 were eligible for the study as a result of them being treated with low-dose TKIs (*n* = 71) and/or having discontinued the treatment (*n* = 25); 9 of them discontinued treatment without previous dose reduction. All patients at diagnosis were in the chronic phase of pathology.

The patient sample included 49 men and 31 women, with a range in age of 27–97 years old (median age = 66 years IQR = 51.7–81.2). The median age when the diagnosis was given was 56 years (IQR =35.7–67.2). The baseline characteristics of all patients are summarised in Table 1.

### 3.2. Clinical Characteristics of TKI Treatment

The initial treatment consisted of first-generation TKI (imatinib) in 60% (48/80) of patients and second-generation TKI (dasatinib or nilotinib) in 40% (38/80) of patients. From the CML patients first treated with a first-generation TKI, 32 of them were then not given TKI; the reasons for the change were resistance (*n* = 12) and intolerance (*n* = 20) to the first-generation TKI.

The most common AEs were gastro-intestinal intolerance in 22.5% of patients, haematological toxicity (mainly thrombocytopenia and anaemia in 18.7%), cardiac toxicity such as heart rhythm disturbances and heart failure in 11.2%, renal failure in 11.2%, osteoarticular pain and myalgia in 7.5%, pleural effusion in 7.5%, dermatological disturbances and oedema in 5% and upper respiratory tract infections (URTI) in 3.7% of patients. Toxicity according to TKI and the line of treatment is summarised in Table 2.

Forty-two patients kept receiving the TKI treatment (52.5%), and thirteen patients died. None of them as a result of CML.

### 3.3. Low-Dose TKIs in Real Life

A total of 71 CML patients underwent dose reduction (88.7%). More specifically, TKI dose reduction occurred in 41% of patients treated with nilotinib, 29.5% treated with dasatinib, 20.5% treated with imatinib, 1.2% treated with bosutinib and 1.2% treated with ponatinib. Dose reduction was caused by AEs in more than half of the cases (56 patients; 78.8%). Before DR, 22 patients achieved MR5, 18 achieved MR4.5, 12 achieved MMR, 5 achieved MR4, 9 achieved DMR (their medical records did not specify the degree of logarithmic reduction) and 5 had not yet achieved MMR. In addition, 15 patients experienced a dose reduction owing to the optimal maintained treatment response: 3 experienced it due to first line imatinib, 2 experienced it due to second line dasatinib and 10 experienced it due to nilotinib (3 were on first line, 6 were on second line and 1 was on third line). Remarkably, six of the patients presented with MR5 at the time of DR due to a maintained response, 4 achieved MR4, 3 achieved MMR and 2 achieved DMR (the degree of logarithmic reduction was not specified in the medical record).

Seven patients started first-line treatment with low-dose imatinib due to comorbidities and the patient’s frailty. In the second-line treatment, four patients started a low-dose treatment with dasatinib (2), imatinib (1) and ponatinib (1). In the third line, three patients started low-dose treatment with nilotinib (2) and bosutinib (1).

Forty-four patients (61.9%) received a 50% dose reduction from the baseline. Overall, 14 (19.7%) patients experienced a >50% reduction in the TKI dose from the baseline, and 13 patients received a DR of <50% (18.3%). Prior to DR, 42 patients (59.1%) had received only a TKI. Out of the other twenty-nine patients who were undergoing a second-line therapy, eight were ultimately switched to a third line of treatment. The median duration of the TKI treatment was 109.1 months (IQR 66.5–155). At the time of DR, 13 patients (18.3%) had not already achieved a deep molecular response (DMR). The median of the DMR duration was 30.7 months (IQR 8–57.7) after the implementation of DR.

### 3.4. Discontinuation Due to Adverse Effects to TKIs in Real Life

Of the 25 discontinuations, 10 were in patients treated with nilotinib (40%), 10 were treated with dasatinib (40%) and 5 were treated with imatinib (20%). A total of 18 discontinuations occurred due to AEs during the TKI treatments (Table 3). Four patients discontinued their treatment due to maintained response, including three patients given a treatment with nilotinib and one who was given dasatinib. Furthermore, two patients who were treated with dasatinib and one treated with nilotinib discontinued their treatment according to their own decisions. The median duration of the TKI before discontinuation was 84.7 months (IQR 27–88).

### 3.5. Outcome

For those patients treated with low doses, the median duration of MRFS was 24.6 months (IQR 6.9–57.6). MMR loss was only experienced by 11 patients (14.1%). This event occurred usually during first-line therapy (63.6%) and affected four patients who were treated with imatinib (36.3%), three patients who were treated with nilotinib and dasatinib (27.2%) and one patient who was treated with bosutinib (9%). From the total number of patients whose dose was reduced to 50% (44 patients), 15.9% of them experienced molecular recurrence. Out of the patients with a >50% reduction, 28.6% stopped responding, and three patients initiated a treatment with reduced doses (21.4%) (Table 4). No patients who underwent a reduction by <50% stopped responding to the treatment.

**Figure 1:**
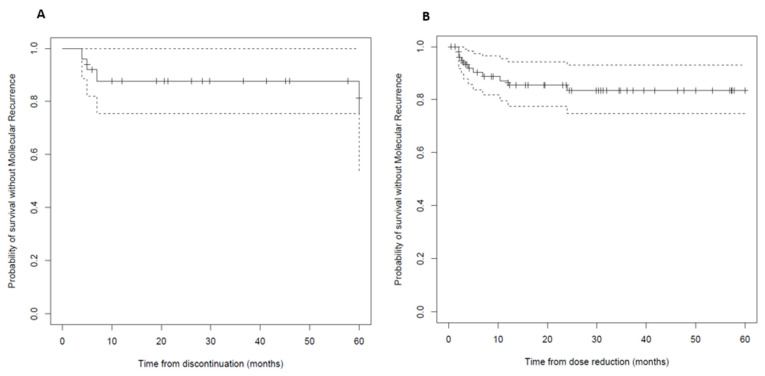
(**A**) Treatment-free remission after TKI discontinuation (Kaplan–Meier estimate). (**B**) Molecular recurrence-free survival after TKI dose reduction (Kaplan–Meier estimate). Solid lines: dose at discontinuation: low dose; dashed lines: CIs 95%.

The median time to molecular recurrence (≥MMR) was 4 months (IQR 2–9.4) (Figure 1), and 7/11 (63.6%) patients experienced an early relapse (within <6 months). The estimated MRFSs to the loss of response or the last follow-up at 12 and 24 months were 67% and 52%, respectively. Patients with longer duration of DMR showed a trend towards a prolonged MRFS.

After molecular relapse, nine patients treated with same TKI had their dose increased, while two patients were switched to an alternative TKI. All patients had an MMR again, except for one patient who was treated with bosutinib doses at 500 mg/day. The treatment of this patient was swapped to ponatinib doses at 45 mg/day.

**Figure 2:**
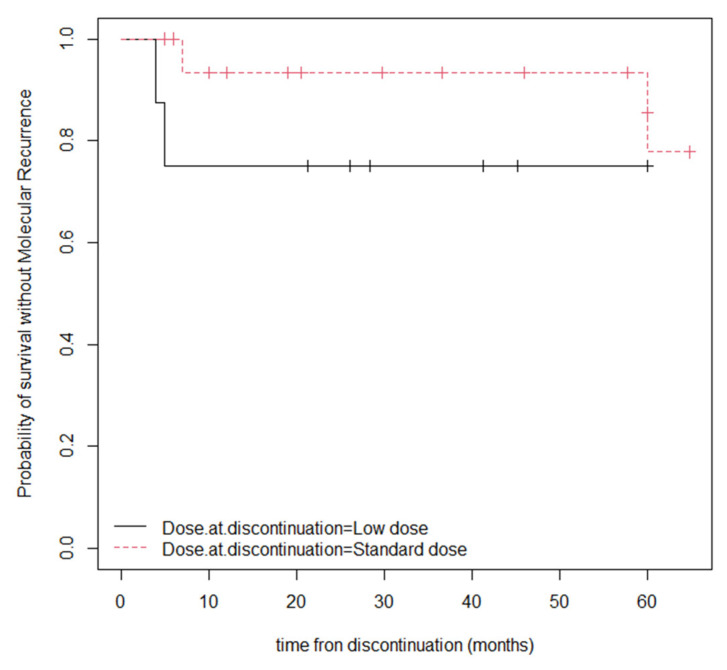
TFR probability according to the TKI dose at discontinuation (Kaplan–Meier).

After a median follow-up of 29.2 months (IQR 12–64.8), after the discontinuation of TKI, 21 (84%) continued to have an MMR. TFR at 12 and 24 months was 80% and 68%, respectively. The median time to molecular recurrence (≥MMR) was 6 months (IQR 4.5–36.5). Four patients who stopped having an MMR (16% of discontinuations) were given the dasatinib treatment, and three were on low-dose dasatinib prior to discontinuation. Two of the people who experienced a molecular recurrence resumed the same dose of TKI treatment, one patient switched to nilotinib and one patient decided not to resume the TKI treatment. The time until TFR among the group of patients who discontinued their TKI after a period of low-dose TKI was 43.1 months, and among those who discontinued taking a standard dose of TKI, it was 16.3 months. In the statistical analysis of the TFR probability, according to the TKI dose at discontinuation, no statistically significant differences were observed (*p*-value = 0.3) between the patients who discontinued the treatment at LD or a standard dose (median survival LD 1.04, CI 95%, 0.88–1.23; median survival at standard dose 2.96, CI 95%, 0.31–1.23) (Figure 2).

**Figure 3:**
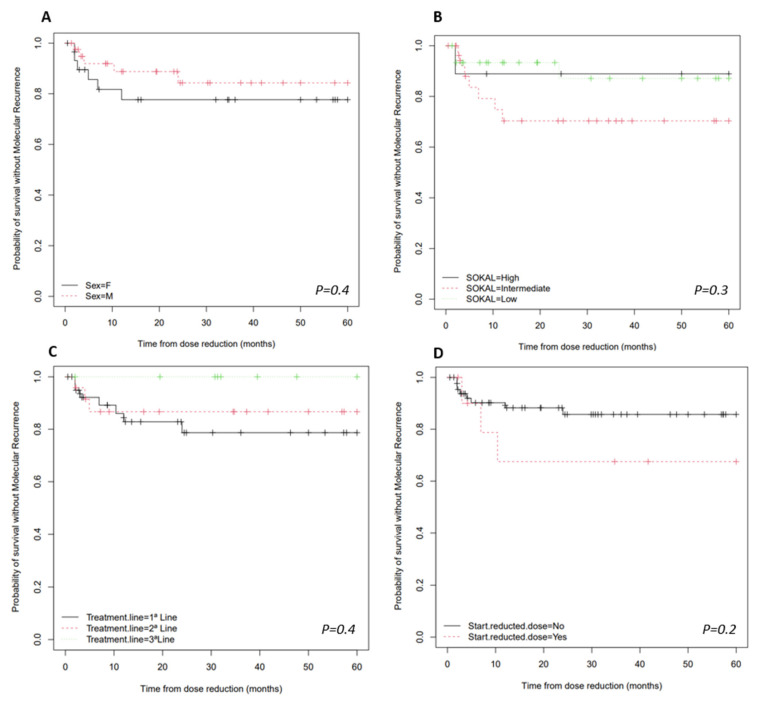
MRFS after TKI dose reduction (Kaplan–Meier estimate). (**A**) MRFS according to sex. (**B**) MRFS according to the SOKAL risk score. (**C**) MRFS according to the treatment line. (**D**) MRFS according to the start reduced dose or standard dose.

In the study of variables that might be associated with molecular recurrence, it was found that it was not affected by any of the variables (Figure 3). The variables examined included gender (*p* = 0.3873), Sokal risk scores (*p* = 0.6737), prior treatment with interferon-alpha (*p* = 0.4457) or hydroxycarbamide (*p* = 0.2884), age at CML diagnosis (*p* = 0.641), the initiation of low-dose therapy (*p* = 0.469) or the median duration of TKI therapy (*p* = 0.6340).

The log-rank test was employed (Figure 3) in order to analyse the relationship between survival probability without molecular recurrence and variables such as sex, Sokal risk scores, the line of therapy where DR occurs or if the patient started the treatment with low or standard doses.

## 4. Discussion

The accurate treatment of CML is critical for the optimal management of patients treated with TKI. This has become increasingly important since the discontinuation of TKI therapy among patients who had a DMR became a therapeutic alternative [14].

A greater selectivity toward neoplastic cells, higher efficacy rates, oral administration and a safer toxicity profile made this therapeutic approach preferable to conventional chemotherapy. Furthermore, high-cytogenetic-risk diseases are much more effectively treated with tyrosine kinase inhibitors. Several trials with patients with CML have already shown that in predetermined conditions (CMR for at least 2 years after ≥3 years of TKI treatment), the production of BCR::ABL1 inhibitor can be discontinued and rechallenged in the case of molecular relapse [15].

Despite the limited number of patients involved in this study, it was revealed that a large percentage of CML patients achieved disease control through the treatment with TKI. However, this is associated with a wide range of chronic side effects, which might require dose adjustments or even the discontinuation of the treatment. The present study shows that in most cases, switching from a first-generation to a second-generation TKI or dose reduction is linked to the appearance of intolerances rather than resistance to the treatment, with gastro-intestinal intolerance standing out from the rest.

Dose optimization minimises the risks of adverse events while maintaining the response to TKI therapy [15]. The number of TFR studies has greatly increased, and data are available from trials conducted after primary or secondary treatments with a second-generation TKI. Trials with nilotinib and dasatinib as a second-line therapy after the patient has shown resistance or intolerance to imatinib, or as a means to optimise deep remission, have shown that TFR is feasible and has similar success to simpler TFR after the use of primary imatinib or second-generation TKI [16,17,18].

It is widely accepted that the standard starting dose of imatinib is 400 mg/day. Some clinical trials using doses higher than 400 mg/day resulted in greater efficacy, but at the expense of a worse side-effect profile, thus deteriorating the patients’ quality of life. There are currently very few data available on the appropriateness of starting imatinib at a dose lower than 400 mg/day, except in frail elderly or polymedicated patients for whom the major concerns are AEs and drug effects [19,20,21].

In some studies, it has been observed that as many as half of the patients can achieve TFR after the discontinuation of a TKI treatment. Moreover, it is observed that molecular recurrence appears within 6 months after discontinuation [12,13]. Factors such as the disease itself or those related to the patient, such as immune status or persistence of CD26+ leukaemic stem cells, may influence the likelihood of achieving and maintaining a TFR. The abrupt discontinuation of TKI therapy without prior dose reduction has been shown to be safe in both clinical trials and real-life studies [22,23,24].

A 5-year-long analysis of a phase III study (DASISION) compared treatments with dasatinib or imatinib among naïve patients with CML. Long-term efficacy and safety outcomes of patients with chronic phase CML treated with these drugs were analysed. At the end of the study, approximately 60% of patients treated with dasatinib and imatinib continued taking their initial treatment. Cumulative rates of major molecular response and molecular responses with a 4.0- or 4.5-log reduction in BCR::ABL1 transcripts from the baseline remained significantly and statistically higher for dasatinib compared to those of imatinib. The rates for progression-free and overall survival at 5 years remained high and similar across both arms [24].

A further retrospective analysis of the DASISION clinical trial revealed that 15% and 28% of the patients underwent dose reduction during treatments with imatinib and dasatinib, respectively. Dose modifications are more frequent for second- and third-generation TKIs. The study again showed that the levels of molecular responses remained higher for dasatinib when daily doses were modified due to AEs. Interestingly, dose reductions did not affect efficacy (MMR rates were not reduced) [25]. The NORDCML006 study also compared the effect of imatinib 400 mg/day with that of dasatinib 100 mg/day on CML patients. Dasatinib dose reductions for AEs were frequent; however, molecular responses were maintained. One imatinib patient progressed to the blastic phase, but no CML related deaths occurred. In conclusion, the data of this study compare favourably with those of the dasatinib registration study, DASISION [26]. In the RERISE study, the imatinib dose was reduced in 19% of the patients, and that of radotinib was reduced in 54% of them. The median age of the patients that participated in these studies was between 45 and 49 years. In our study, the median age was 66 (IQR 51.7–81.2). Therefore, it seems that elderly patients experience TKI dose reduction more frequently than younger patients do, which is in part due to the comorbidities of these patients [27].

In the UK, the doses given to DESTINY study patients were reduced by half of the standard dose for 12 months prior to treatment discontinuation. Most patients in this study were treated with imatinib as a first line. Even though the technique seems to be appropriate, maintaining standard pharmacological doses is not always possible due to the fact that the appearance of intolerance to the treatment is frequent. This study aimed to evaluate the effects of treatment de-escalation prior to the discontinuation of the treatment by patients who experienced MR4 or greater and patients who experienced MMR, but not MR4. During the time, of those patients who experienced DR, only 7% experienced molecular recurrence, and all of them received imatinib. It was also observed that the probability of suffering molecular recurrence when dose de-escalation was performed had no relation with the duration of the treatment, age, sex, weight or BCR::ABL1 type [28].

As a result of the study, it was observed that treatment de-escalation is feasible for patients that have reached a stable level of MR4 or deeper remission. It was also shown that the absence of stable MR4 is a factor to be considered prior to the discontinuation of treatment. All patients who relapsed quickly were able to regain an MMR or had an improved response four months after treatment resumption at standard doses.

During the time of treatment de-escalation, there was a saving in the cost of the treatment. In the UK, the cost of imatinib is proportional to the dose used, unlike nilotinib or dasatinib, where there is no price difference. From this, it was concluded that cost savings were achieved for those patients who relapsed and subsequently resumed standard dosing [28].

The study performed by Claudiani et al. [29] showed that most patients who underwent DR with imatinib were able to stay at the MR3 level for both of the reduced doses (300 and 200 mg daily). At the time of DR, the level of molecular response was MR4 in 204 of 298 cases (68.4%). The reasons for DR include any degree of adverse event deemed to be significant by the clinician or pre-emptive DR at the time of the introduction of a subsequent TKI due to intolerance to the previous TKI. Imatinib was given as first line in 89 of 90 cases (98.8%), whereas a second-generation TKI was ≥2 line in 177 of 208 cases (85%). In 274 of 298 cases of DR (91.9%), MR3 was maintained on the LD TKI at a median follow-up of 27·3 months.

The NILO-RED study [30] is also consistent with these results. Herein, the outcome of changing nilotinib from a twice daily to a once daily regimen was reported, resulting in DRs of 25–50% in 67 patients who had achieved MR3 for a median of 25 months, and the 12 months probability of survival without unconfirmed MMR loss was 97%.

Claudiani and NILO-RED’s findings can be compared with our study, where imatinib was also used as a first-line treatment in most of the patients. In the present study, the DR in 78.8% of the patients was due to AE. Regarding the outcomes of the DR, MMR loss was only experienced by 11 patients (14.1%), which is consistent with the results of the studies mentioned above and is hopeful in terms of the ability to achieve TFR after DR. Note that at the time of dose reduction, most patients had already achieved an MMR. This outcome is consistent with the results of previous studies, which not only suggest that performing dose reductions is safe, but also design an optimal therapeutic strategy.

Another study [12] on the use of a low-dose TKI and its impact on TFR showed that the patients maintained a molecular response (median follow-up 29.2 months). No association was found between variables such as sex, risk score, previous interferon, BCR::ABL1 transcript types, the number and type of TKIs used before cessation of treatment, the degree of DMR or the median duration of therapy with TKIs with the outcome of TFR. Improved TFR was observed after dose reduction by AEs and after the prolonged duration of DMR. An even lower percentage of molecular recurrence was observed in patients with reduced doses compared to that of those given standard doses [12].

Several studies using intermittent treatment with an TKI drug showed that sometimes patients with a good response are overtreated. An example of this can be seen in the Italian study, INTERIM, in which CML patients over 65 years of age treated every other month with imatinib doses of 400 mg/day experienced improved DMRs and also maintained their MMRs [31,32].

Results such as those in this study and the ones we have mentioned before support the fact that even though the overall TFR response rates are not higher in studies using second-generation TKIs as a first-line treatment, the rapidity of achieving a deep molecular response might be key to make a clinical decision. Variables such as reducing the duration and exposure to the treatment and potential toxicities associated with the treatment should be considered. If TFR was more widespread and globally achievable, the perspective on toxicity could be changed as the risks of short- and long-term treatment are different. Critical thinking regarding the benefits and risks of limiting the duration of treatment for CML patients would be considered to evolve with respect to long-term toxicity [33,34].

## 5. Conclusions

An increasing number of patients are suitable candidates for the optimisation of the dose of TKI therapy. Although several studies of TKI cessation have been reported, little is known about the feasibility of treatment de-escalation among patients with stable molecular responses. However, there are still more studies that need to be conducted to meet the unmet needs for adequate treatment of CML. These include minimising toxicity and the risk of disease progression, as well as trying to boost initial response with safer and more effective approaches for those patients who are resistant or intolerant to TKI treatments.

The findings of this study demonstrate that most patients who are given a reduced TKI dosage in the presence of MMR maintain their response. These results are in line with all similar studies analysed herein. Consequently, it seems feasible to administer reduced doses of TKI to patients with chronic myeloid leukaemia and a stable MMR. Indeed, it has been observed a positive response regarding the improvement of adverse events associated with the treatment. In other words, thanks to DR, many patients with stable responses might be able to maintain their responses on lower TKI doses with a lower risk. Furthermore, DR allows us to personalise a patient’s treatment and establish an effective dose for each patient according to their characteristics. Similarly, the study also shows that it is possible to discontinue a treatment after dose reduction and continue to maintain the MMR. Stopping TKI therapy after achieving a sustained deep molecular response is an emerging treatment goal for patients with CML in the chronic phase.

All medical aspects of CML patients should be considered as a whole, and the strategy implemented should be taken into account before proceeding with the treatment. The use of DR is not established in clinical practice, but in patients who are intolerant to treatment or where prolonged use of standard doses would compromise safety or adherence to the treatment, DR should be considered as it is a safe option that does not compromise, in most cases, the achievement of a DMR.

In addition, de-escalation and discontinuation are also associated with substantial financial savings, and this could be very useful to optimise resources and promote greater efficiency in the use of TKI. More studies are still needed, but the results reported so far are promising and call for further research in this area. These recommendations could pave the way for the implementation of optimised dosing regimens, and thereby, contribute to an improved and sustainable CML therapy.

## Figures and Tables

**Table 1:** Baseline demographics and clinical characteristics of CML patients.

M/F	49/31
Median age	66 (IQR 51.7–81.2)
Median age at diagnosis	56 (IQR 35.7–67.2)
BCR::ABL1 transcript type, *n* (%)	
p210	13 (16.2)
p190	2 (2.5)
Previous treatments of TKI, *n* (%)	
Hydroxycarbamide	71 (88.7)
Interferon-alpha	5 (6.2)
Cytarabine	5 (6.2)
Busulfan	1 (1.2)
Comorbidities, *n* (%)	
No	37 (46.2)
HBP	18 (18.8)
DM	10 (18)
Hypercholesterolemia	8 (10)
Endocrine disorders	7 (7.8)
Autoimmune diseases	2 (2.5)
Neoplasia	3 (3.7)
Sokal, *n* (%)	
Low	37 (46.2)
Intermediate	31 (38.7)
High	10 (12.5)
NA	2 (2.5)
First Line, *n* (%)	
Imatinib	48 (60)
Nilotinib	20 (25)
Dasatinib	12 (15)
Second Line, *n* (%)	
No	36 (45)
Imatinib	4 (5)
Nilotinib	21 (26.2)
Dasatinib	18 (22.5)
Ponatinib	1 (1.2)
Third Line, *n* (%)	
No	66 (82.5)
Nilotinib	4 (5)
Dasatinib	7 (8.7)
Ponatinib	2 (2.5)
Bosutinib	1 (1.2)
Duration of TKI	109.1 (IQR 66.5–155)
Duration of low-dose TKI (months, median)	24.6 (IQR 6.9–57.6)
Duration TKI before discontinuation (months, median)	84.7 (IQR 27–88)
Maximum response in patients with low dose TKI, *n* (%)	
MMR	11 (15.4)
MR4	2 (2.8)
MR4.5	18 (25.3)
MR5	29 (40.8)
CMR	11 (15.4)
MR at TKI discontinuation, *n* (%)	
MMR	2 (8)
MR4	1 (4)
MR4.5	8 (32)
MR5	11 (44)
CMR	3 (12)

HBP: high blood pressure; DM: Diabetes mellitus; NA: not available; MMR: major molecular response; MR4: molecular response log 4; MR4.5: molecular response log 4.5; MR5: molecular response log 5; CMR: complete molecular response; MR: molecular response.

**Table 2:** Number of patients with toxicity by medication and line of treatment.

Toxicity	Imatinib			Nilotinib			Dasatinib		
	1st Line	2nd Line	3rd Line	1st Line	2nd Line	3rd Line	1st Line	2nd Line	3rd Line
Cardiac	1			3	3		1	1	
Dermatological	2				2				
Digestive	8	1		1	1		3	2	1
Edema	2				2				
Haematological	5			4	2		1	4	
Hepatic				1	1	1			
Myalgia	2								
Osteoarticular	2				1				1
Pleural effusion							4	1	1
Pulmonary					1		1		
Renal failure	7				1		1		
URTI							1		2

**Table 3:** Main adverse effects associated with TKI discontinuation among patients.

Toxicity	TKI (*n*)
Cardiac	Nilotinib (3)
Dermatological	Nilotinib (1)
Digestive	Imatinib (3) Nilotinib (1) dasatinib (1)
Edema	Nilotinib (1)
Haematological	Dasatinib (2)
Myalgia	Imatinib (1) Nilotinib (1)
Pleural effusion	Dasatinib (1)
Pulmonary thromboembolism	Nilotinib (1)
Renal failure	Imatinib (2)

**Table 4:** Treatment characteristics in low-dose-receiving patients who stopped having an MMR.

TKI	Low Dose (mg)	Reduction (%)	Line	Age	Month Loss MMR	Start Reduced Dose	Therapeutic Management
Imatinib	100	75 *	1st Line	90	3	Yes	Dose increased to 300 mg/day
Imatinib	100	75 *	1st Line	92	6.9	Yes	Dose increased to 300 mg/day
Imatinib	100	75 *	1st Line	93	10.4	Yes	Dose increased to 300 mg/day
Imatinib	200	50	1st Line	38	24	No	Dose increased to 400 mg/day
Nilotinib	300	50	1st Line	83	12	No	Dose increased to 600 mg/day
Nilotinib	300	50	1st Line	41	2	No	Switched to 600 mg/day dasatinib
Nilotinib	300	50	1st Line	43	2	No	Dose increased to 600 mg/day
Dasatinib	50	50	2nd Line	36	4	No	Switched to 600 mg/day nilotinib
Dasatinib	50	50	2nd Line	79	4.9	No	Dose increased to 100 mg/day
Dasatinib	50	50	2nd Line	60	2	No	Dose increased to 100 mg/day
Bosutinib	100	80	4th Line	71	2.6	No	Dose increased to 500 mg/day

* Percentage dose reduction if the patient was given a treatment at a standard dose of 400 mg/day.

## Data Availability

Not applicable.
